# Rapid and damage-free outgassing of implanted helium from amorphous silicon oxycarbide

**DOI:** 10.1038/s41598-018-23426-y

**Published:** 2018-03-22

**Authors:** Qing Su, Hepeng Ding, Lloyd Price, Lin Shao, Jonathan A. Hinks, Graeme Greaves, Stephen E. Donnelly, Michael J. Demkowicz, Michael Nastasi

**Affiliations:** 1 Nebraska Center for Energy Sciences Research, University of Nebraska-Lincoln, Lincoln, NE 68583-0857 USA; 2 Department of Materials Science and Engineering, Texas A&M University, College Station, TX 77843-3128 USA; 3 Department of Materials Science and Engineering, Massachusetts Institute of Technology, Cambridge, MA 02139 USA; 4 Department of Nuclear Engineering, Texas A&M University, College Station, TX 77843-3128 USA; 5 School of Computing and Engineering, University of Huddersfield, Huddersfield, HD1 3DH UK; 6 Department of Mechanical and Materials Engineering, University of Nebraska-Lincoln, Lincoln, NE 68583-0857 USA

## Abstract

Damage caused by implanted helium (He) is a major concern for material performance in future nuclear reactors. We use a combination of experiments and modeling to demonstrate that amorphous silicon oxycarbide (SiOC) is immune to He-induced damage. By contrast with other solids, where implanted He becomes immobilized in nanometer-scale precipitates, He in SiOC remains in solution and outgasses from the material *via* atomic-scale diffusion without damaging its free surfaces. Furthermore, the behavior of He in SiOC is not sensitive to the exact concentration of carbon and hydrogen in this material, indicating that the composition of SiOC may be tuned to optimize other properties without compromising resistance to implanted He.

## Introduction

The limited resistance of current engineering materials to radiation damage is a key factor restricting the design of next generation nuclear reactors^1^. Consequently, much effort has been invested into averting a wide range of radiation-induced degradation phenomena, such as swelling^2^, embrittlement^3^, and accelerated corrosion^4^. Materials fit for service under the harsh operating conditions of future nuclear reactors must be simultaneously tolerant to each of these damage mechanisms while remaining economical. This requirement motivates investigations of amorphous silicon oxycarbide (SiOC): an easy-to-synthesize material^5^ that exhibits remarkable insensitivity to ion-induced displacement damage^6,7^ in addition to high temperature stability, with crystallization temperatures >1300 °C as well as creep and corrosion resistance^5,8–10^. Here, we show that SiOC is also insensitive to another form of radiation-induced damage: degradation due to implanted helium (He). This finding demonstrates that SiOC is simultaneously resistant to several forms of radiation-induced degradation, markedly elevating its potential for use in advanced nuclear reactors.

Radiation damage in materials may be classified broadly into two categories: displacement damage and degradation induced by the introduction of impurities. The former occurs when an atom in the material displaces permanently from its initial location due to a collision by an impinging particle. In crystalline solids, atom displacements generate vacancies and interstitials^11^ while in network-bonded amorphous solids, like SiOC, they produce coordination defects such as dangling bonds^12^. While displacement damage disrupts the atomic-level structure of the material, it is potentially recoverable without long-range mass transport, e.g. through vacancy-interstitial recombination in crystals^13^ or relaxation/reconstruction of radiation-affected regions in amorphous solids^14^. Indeed, numerous strategies for mitigating displacement damage have proven effective^15^.

By contrast, radiation damage through the introduction of impurities is more difficult to mitigate because it changes the composition of the material itself. This form of damage occurs due to transmutation of atoms in the material^16^ or through high-energy implantation of foreign species^17^. Certain impurities—such as hydrogen—may eventually diffuse out of the material, depending on temperature and the density of trap sites^18^, affording an opportunity for recovery of damage. However, others—notably, noble gases such as He, neon, xenon, or krypton^19–22^—form stable precipitates and become immobilized. Some material-design strategies have aimed to delay this form of damage by spreading the impurities over larger numbers of smaller precipitates^23^. This approach, however, cannot avert damage in the long run as continued introduction of additional impurities eventually saturates all traps and precipitation sites, whereupon the usual damage processes resume.

The resistance of SiOC to He-induced damage demonstrated here is exceptional in this context. We find that, unlike in most solids^19,20^, He implanted into SiOC does not form precipitates but remains in solution and undergoes rapid diffusion. Indeed, we observe complete outgassing of implanted He from SiOC in the time span of our experiments. Moreover, the egress of He from SiOC does not produce any detectable surface damage, unlike in other nuclear materials^24,25^. These findings indicate that He-induced damage in SiOC may be averted entirely, rather than merely delayed. Finally, using first-principles modeling, we demonstrate that the behavior of He impurities in SiOC is comparable regardless of their location relative to different elements in the material, indicating that SiOC is resistant to He-induced damage over a wide range of compositions. Therefore, composition may be tuned to minimize other forms of radiation damage in SiOC without compromising immunity to He-induced damage.

## Results

### *Ex-situ* He implantation and characterization of He content

To investigate the response of SiOC to He impurities, we use a combination of ion beam analysis as well as both *ex situ* and *in situ* ion implantation and transmission electron microscopy (TEM). These techniques allow for rapid materials testing without resorting to irradiation in a nuclear test reactor. However, they require small-scale samples of high perfection. We use radio frequency sputtering to synthesize thin-film samples of SiOC (see Methods). These samples consist of a ~300 nm-thick SiOC surface layer deposited atop a ~300 nm-thick amorphous SiO2 (a-SiO2) on a thick Si substrate.

We implant He into these samples at room temperature and at 600 °C to doses that give rise to peak implanted He concentrations of 10 atom% (see Methods). The 50 keV He^+^ ion beam used for implantation impinges upon the sample surface at a 45 degree angle. Approximately half of the He is implanted into the SiOC layer while the remainder comes to rest in the underlying a-SiO_2_ layer (Supplementary Fig. S1). The ion beam causes ~6 displacement per atom (dpa) peak displacement damage. After implantation, we check for He retention in our samples using proton backscattering (p-BS, see Methods). For comparison, we also carry out an identical set of He implantations and p-BS measurements on a Si wafer with ~300 nm-thick a-SiO_2_ native oxide, but no deposited SiOC layer.

**Figure 1:**
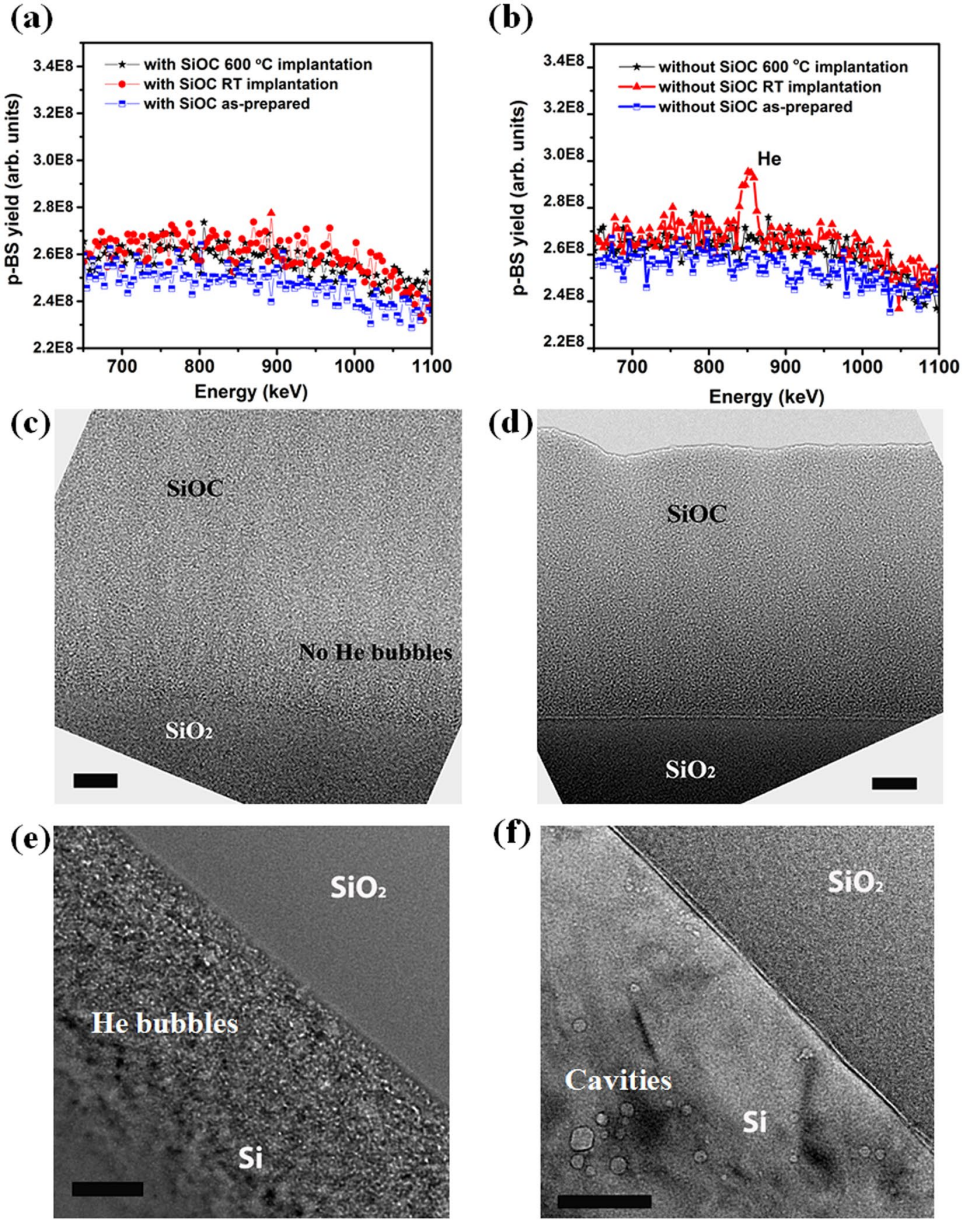
p-BS spectra for samples (**a**) with and (**b**) without a deposited SiOC layer. Spectra for room temperature and 600 °C implantation to a peak He concentration of 10 atom% are shown as are baseline spectra for as-prepared samples prior to any He implantation. TEM micrographs of samples implanted with He to a peak concentration of 10 atom%: SiOC-capped samples after (**c**) room temperature and (**d**) 600 °C implantation. *Scale bar:* 20 nm. No He bubbles were observed. Samples with no SiOC layer after (**e**) room temperature and (**f**) 600 °C implantation; He bubbles or cavities were present. *Scale bar:* 50 nm.

Figure 1a and b compare p-BS spectra for samples with and without deposited SiOC, each implanted to a peak He concentration of 10 atom%. Spectra for both implantation temperatures (room temperature and 600 °C) are shown. The figure also shows baseline spectra measured prior to any He implantation, demonstrating that both sample types are He bubbles-free in their as-prepared state. The featureless p-BS spectra for the SiOC-capped samples shown in Fig. 1a demonstrate no detectable He bubbles in these samples after implantation at both room temperature and at 600 °C. By contrast, Fig. 1b shows an unmistakable He peak after room temperature implantation into the sample with no deposited SiOC layer. This peak is absent in the same sample type after He implantation at 600 °C.

The spectra in Fig. 1 demonstrate egress of implanted He from SiOC within the time required to commence p-BS measurements (<1 hour). This finding demonstrates that SiOC does not retain implanted He above the p-BS detection limit and allows He to outgas rapidly. Consistent with previous studies^26,27^, our findings also confirm lack of He retention in the a-SiO_2_ oxide layer beneath the deposited SiOC. Since He escapes from a-SiO_2_, the He observed after room temperature implantation in samples with no SiOC layer is due to retention within the pure Si underlying the surface oxide. Lack of He in the same sample type after implantation at 600 °C indicates that, at this temperature, implanted He also escapes from pure Si.

To corroborate the deductions described above, we characterize the He-implanted samples using TEM (see Methods). Figure 1c and d show that neither SiOC nor a-SiO_2_ layers contain any He bubbles after room temperature or 600 °C He implantation to 10 atom%. This observation is consistent with implanted He remaining in solution in both materials prior to outgassing. Using high resolution TEM and selected area diffraction (Supplementary Fig. S2), we also confirm that both materials remain amorphous with no evidence of crystallization or phase changes in either. These results are consistent with previous findings that irradiation only results in local structural rearrangement of SiOC: a decreased number of Si-O bonds and an increased number of Si-C and C-O bonds^28,29^.

By contrast, pure Si contains copious nanoscale He precipitates after room temperature implantation, as shown in Fig. 1e. Previous investigations also observed such He precipitation in Si^30^. After 600 °C implantation, Si contains a low density of larger cavities, shown in Fig. 1f. Since p-BS finds no evidence of He in this sample, we conclude that these cavities do not contain detectable quantities of He and may have formed *via* condensation of supersaturated vacancies generated by atom displacements. Alternatively, they may also be remnants from He precipitates that formed initially, but from which He subsequently escaped.

### *In-situ* He implantation

**Figure 2:**
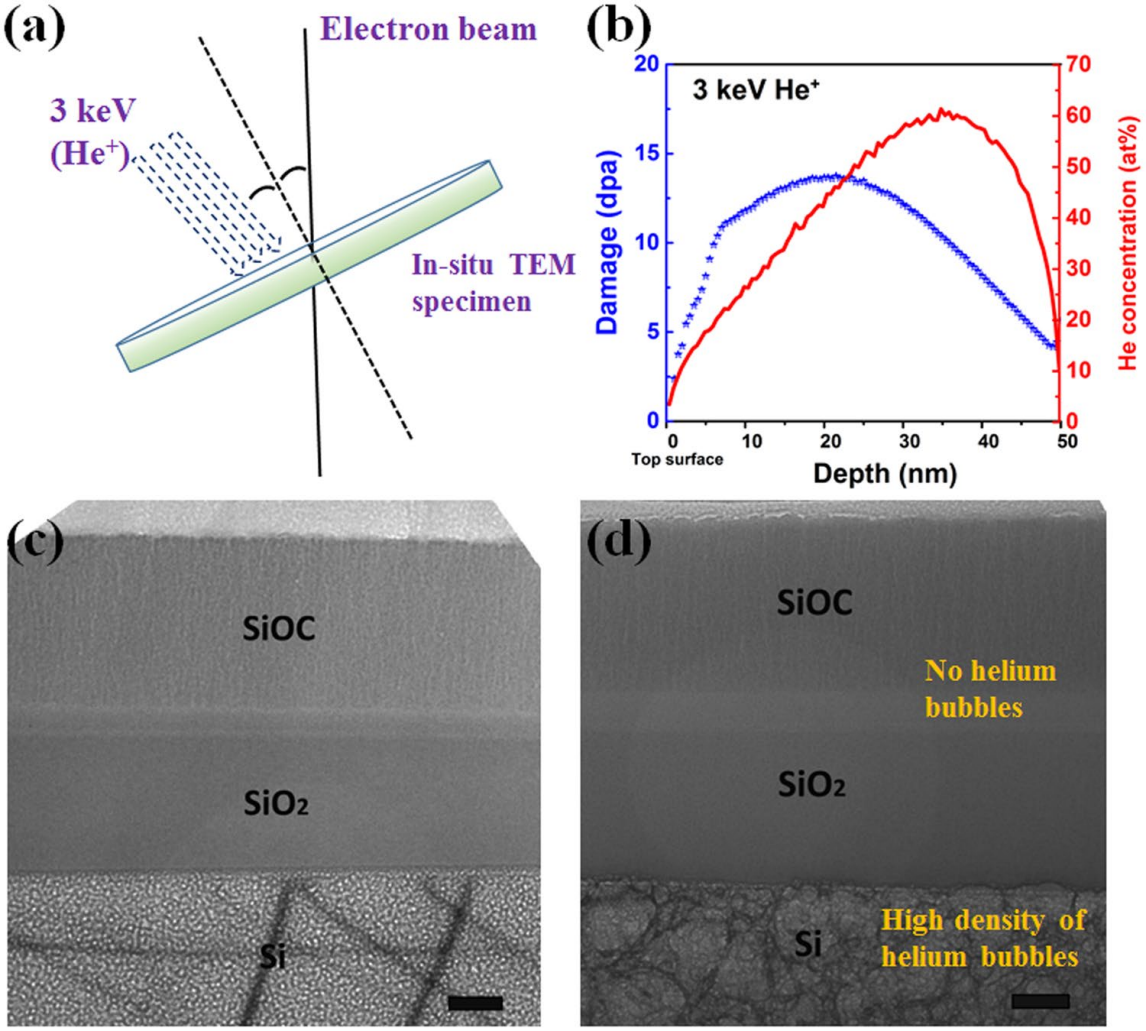
(**a**) Schematic of *in situ* He implantation and (**b**) depth profile of the resulting displacement damage and He concentration. Bright-Field TEM micrographs of SiOC-capped samples after *in situ* He implantation to peak He concentration of (**c**) ~90 atom% at 107 K and (**d**) ~60 atom% at room temperature. To enhance bubbles contrast, the images use ~1000 nm under-focus. *Scale bar*: 100 nm.

To determine whether there is any transient formation of He precipitates in SiOC or a-SiO_2_, we conduct *in situ* TEM observations during high dose, room temperature He implantation at the MIAMI-1 Facility at the University of Huddersfield^31^ (see Methods). To increase the likelihood of observing precipitates, we also repeat these experiments at cryogenic temperatures (107 K), where He mobility (and hence also the outgassing rate) is expected to be significantly lower than at room temperature. Figure 2c and d show TEM micrographs of the samples after He implantation to a peak dose of 90 and 60 atom%, respectively. Unlike the previously described *ex situ* implantation experiments, in our *in situ* experiments, the He beam is nearly perpendicular to the field of view in Fig. 2c and d, i.e. parallel to the sample surface and the SiOC/a-SiO_2_/Si interfaces, as illustrated in Fig. 2a. Thus, each layer shown in Fig. 2 receives an approximately identical dose of He during the experiment. At no point during these experiments did we observe any He precipitate formation in either SiOC or a-SiO_2_, demonstrating that He indeed outgasses continuously from these materials without ever precipitating out of solution, even at cryogenic temperature. By contrast, a high density of He precipitates forms in Si at both 107 K and room temperature.

**Figure 3:**
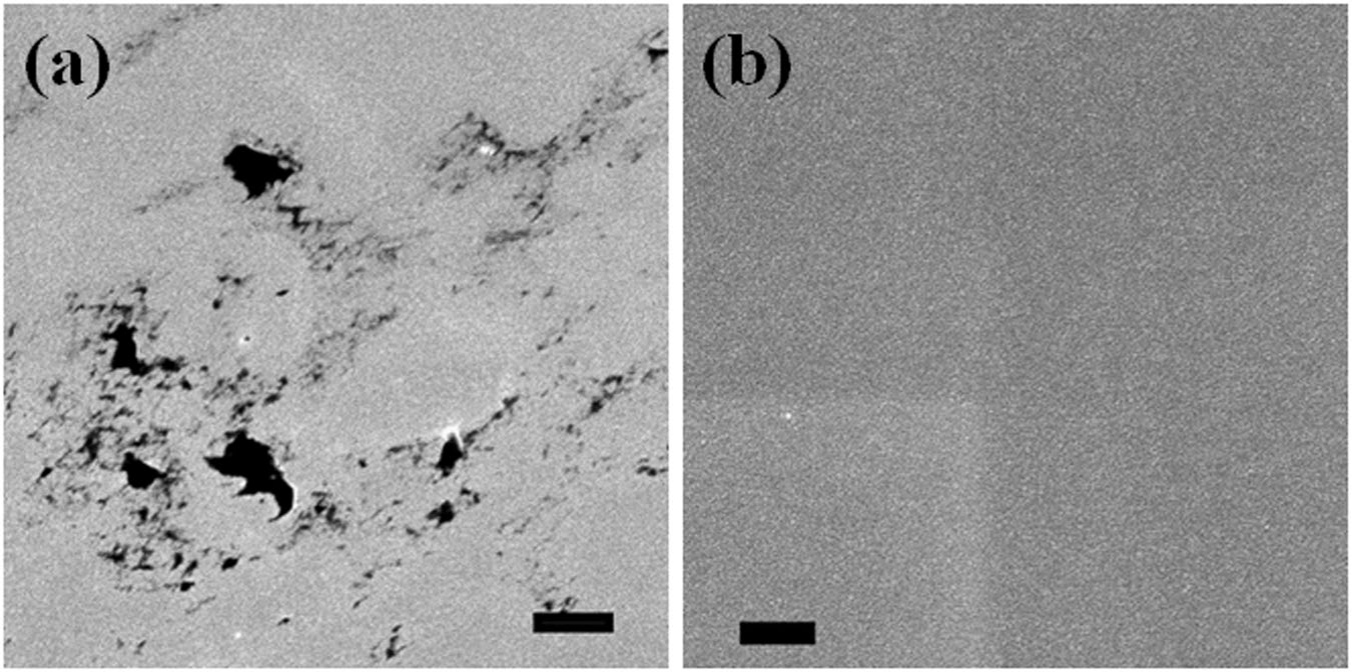
(**a**) SiCN film after 7.4 × 10^21^ ions/m^2^ *ex situ* He implantation at room temperature. *Scale bar*: 2 μm. Surface cracks and voids are present. (**b**) SEM micrograph of a SiOC surface after 1.6 × 10^21^ ions/m^2^ *ex situ* He implantation at room temperature showing no surface damage. *Scale bar*: 1 μm.

The foregoing results demonstrate that outgassing of He from SiOC occurs concurrently with He implantation. This process is facilitated by the lack of He precipitate formation over a wide range of temperatures, from 107 K to 600 °C (873 K), and up to high doses of implanted He. In addition, unlike in other materials where surface blisters or cracks are formed upon He release (Fig. 3a and others)^24,25,32^, He outgassing from SiOC is not accompanied by any surface damage. Scanning electron microscopy imaging of SiOC surfaces after 1.6 × 10^21^ ions/m^2^ *ex situ* He implantation at room temperature shows no evidence of blister formation, cratering, porosity, or any other form of surface morphology evolution (Fig. 3b). It is consistent with outgassing of individual He atoms, as expected given that He remains in solution throughout the entire implantation process. These results indicate the mechanism of He outgassing in SiOC and SiO_2_ is qualitatively distinct from that of crystalline metals and ceramics: He desorption in the latter is due to the coalescence of He bubbles and their breaking out through sample surfaces.

### DFT simulation

To better understand the physical mechanisms underlying the behavior of He in SiOC, we calculate He interstitial formation and migration energies as well as He dimer interaction energies using density functional theory (DFT, see Methods). Formation energies characterize He solubility, migration energies He diffusivity, and dimer interaction energies which determine the tendency of He to cluster into precipitate nuclei. In crystalline solids, each of these quantities may be calculated exactly from a single atomic configuration. However, in amorphous materials, such as SiOC or a-SiO_2_, every atomic site has a distinct local structure, giving rise to a range of site-specific defect energies. Therefore, we compute the quantities of interest at several different locations within our atomistic models and report their average values and standard deviations.

**Figure 4:**
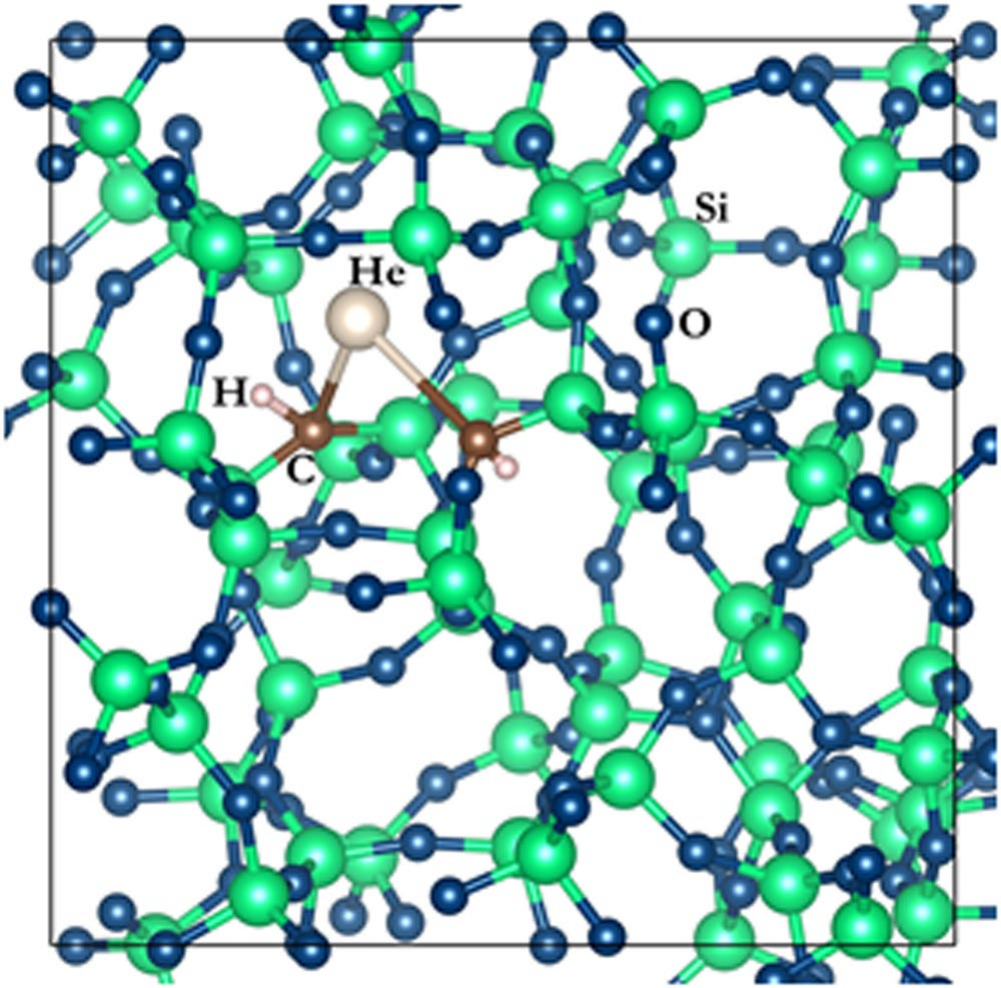
An example of an atomic configuration containing one He atom in the vicinity of two C-H complexes, each of which has been inserted in place of one of two nearest-neighbor O atoms in an atomic model of a-SiO_2_.

Previous investigations have shown that reliable atomistic models of SiOC must contain approximately 1500 atoms: a relatively large number for DFT calculations^12,33^. Thus, to optimize use of computational resources, we do not perform our calculations directly on SiOC models. Rather, we make use of the short range of chemical interactions between He and other atoms^34^ to perform all of our calculations in carbon(C)-doped models of a-SiO_2_. In this approach, we replace two nearest neighbor O atoms in an a-SiO_2_ model with C atoms or—to mimic atomic environments in hydrogenated SiOC—with C-hydrogen(H) complexes. Figure 4 shows an example of such an atomic configuration containing one He interstitial with two nearby C-H complexes. As a baseline for comparison, we also calculate the energies of interest in undoped a-SiO_2_.

To select He interstitial insertion sites, we observe that crystalline SiO_2_, a-SiO_2_, and SiOC are all covalent network-bonded solids whose structures may be viewed as consisting of irreducible rings of atoms^35,36^. In most forms of crystalline SiO_2_, all rings are 6-membered: they contain six Si atoms, each linked to two neighboring O atoms. However, a-SiO_2_ consists of rings with three to nine members, with rings between five and eight members being the most common. We found that there are no stable He interstitial sites in the vicinity of 3- and 4-membered rings and that He occupies 5-membered rings with relatively-high formation energies (~0.6 eV). We therefore exclude from consideration interstitial sites near rings with three to five members, focusing instead on sites near rings with six to nine members.

Table 1 presents the results of our calculations for all three energies of interest in a-SiO_2_, H-free SiOC, and hydrogenated SiOC (SiOC-H). Mean He interstitial formation energies in all three materials are marginally positive with values close to those of low-density SiO_2_ polymorph β-cristobalite (0.035 eV)^37^ and comparable to the thermal energy at room temperature (k_B_T ≈ 0.025 eV). The standard deviations of He interstitial formation energies are a factor of ~3 larger than the means, causing the He interstitial formation energy distributions to overlap zero. These findings indicate that—at room temperature and above—He absorption into all three materials is thermodynamically neutral: it incurs no energy penalty and is therefore neither exothermic nor endothermic. Consequently, no matter the He concentration, there is neither a driving force for He to precipitate nor an opportunity for He to become trapped by binding to low-energy sites. This state of affairs is ideal for He to remain in solution while diffusing rapidly. By contrast, the formation energy of He in high-density SiO_2_ polymorph α-quartz is ~0.5 eV^37^ while in pure, diamond-cubic Si it is ~1 eV^38^, giving rise to a definite solubility limit and the possibility of trapping at high-volume sites.

**Table 1:** He defect energies calculated using DFT.

Material	Formation energies		Migration energies		Dimer binding energies	
	Average ± standard deviation (eV)	Number of averaged values	Average ± standard deviation (eV)	Number of averaged values	Average ± standard deviation (eV)	Number of averaged values
a-SiO_2_	0.031 ± 0.075	52	0.347 ± 0.399	30	−0.048 ± 0.041	22
SiOC	0.028 ± 0.077	23	0.143 ± 0.128	10	−0.027 ± 0.005	20
SiOC-H	0.013 ± 0.043	18	0.183 ± 0.169	7	0.009 ± 0.043	15

He migration energy distributions in SiOC and SiOC-H are indistinguishable within the variance of our calculations. Their average values are comparable to He interstitial migration energies in β-cristobalite (0.13 eV)^37^ (as well as in several transition metals^39–41^), consistent with rapid He diffusion in these materials, even at cryogenic temperatures. The average migration energy for He in a-SiO_2_ is approximately double that of SiOC and SiOC-H and comparable to that of α-quartz (~0.40 ± 0.12 eV)^37^, suggesting lower He diffusivity over a wider range of temperatures. Since all the materials investigated here are amorphous, the true He diffusivity in them does not depend on isolated migration energies alone, but rather on the rate-limiting barrier along representative volume element-spanning diffusion paths. Thus, the foregoing results should be understood as providing estimates of lower and upper bounds to effective He interstitial migration energies. In SiOC and SiOC-H, both of these bounds are substantially lower than the migration energy in diamond cubic Si, which we determine to be 0.74 eV.

It bears mentioning that excess free volume has been found to have a marked influence on impurity diffusion in some amorphous solids^42^. However, in silicates, its effect appears to be secondary compared to the effect of network bonding topology. For example, Lin *et al*.^37^ investigated the effect of volumetric dilatation and compaction on the formation and binding energies of He interstitials in crystalline SiO2 polymorphs alpha-quartz and beta-crystobalite. They found the effect of these volume changes on defect energies to be negligible by comparison to the differing crystal structures of these polymorphs. Therefore, we expect that the He defect energies presented in Table 1 remain representative of SiOC even if the excess free volume of the material changes. On the other hand, phase transformations, such as crystallization, may have a pronounced effect on He defect energies in SiOC.

Finally, the He interstitial dimer binding energies in a-SiO_2_ and SiOC are marginally negative with average values comparable to those of α-quartz (−0.03 eV)^37^ and diamond-cubic Si (−0.025 eV)^38^. These values may lead to a weak tendency for He interstitials to cluster at cryogenic temperatures in these materials, but at higher temperatures the concentration of dimers is likely to be low. The dimer binding energy in SiOC-H is marginally positive and comparable to that of β-cristobalite (0.005 eV)^37^. Thus, there is no driving force for He interstitials to bind at any temperature in these materials.

## Discussion

We have shown that SiOC is immune to He-induced damage due to lack of He precipitation and rapid He diffusion in this material. This finding is especially significant in light of the already impressive resistance of SiOC to several other forms of damage, including displacement damage^6^, corrosion, and creep^8,43^. This confluence of properties markedly elevates the potential of SiOC for use in future reactor designs. The promise of SiOC is not limited to its performance as a stand-alone material, but also extends to its use as a component in multiphase composites. For example, recent investigations of Fe/SiOC laminates show that the SiOC phase reduces accumulation of He in the Fe component. Additionally, the crystalline/amorphous interfaces in such composites further reduce displacement damage by absorbing defects from the crystalline Fe^44,45^.

The DFT calculations and experiments we presented are consistent with each other: both indicate no He precipitation and rapid He outgassing *via* diffusion of isolated He atoms in a-SiO_2_, SiOC, and SiOC-H. These behaviors contrast with pure, diamond-cubic Si (as well as most metals and ceramics)^19,46^, where He interstitial formation and migration energies are relatively higher, providing a driving force for precipitation of supersaturated He and slowing He diffusion. We ascribe these differences to the relatively-open continuous random network (CRN)^47^ structure of a-SiO_2_, SiOC, and SiOC-H, compared to the more compact structure of most other solids. Moreover, since all three CRN materials are insulators with strong, covalent bonding, the electronic charge densities in them are tightly localized, yielding low electron densities at interstitial sites. By contrast, Si is a semiconductor with a more de-localized internal electronic charge distribution, giving rise to higher electron densities at interstitial sites. Thus, insertion of a He interstitial into Si leads to greater electron charge redistribution than in the CRN materials, resulting in higher formation energies. (Supplementary Fig. S3)

Our calculations furthermore provide insights into the effect of C and H on He behavior in the CRN materials: C reduces He migration energies while H reduces He interstitial formation and dimer binding energies. However, in both cases, their effect is small, indicating that the resistance of SiOC to He-induced damage does not depend crucially on composition. This finding is important for the continued development of radiation-resistant SiOC materials, as it suggests that their composition may be tuned to optimize other properties—such as thermal stability^33^ or resistance to displacement damage^12^—without affecting their immunity to He. However, if the C concentration increases so much that the CRN structure begins to break down, we expect that the behavior of He in SiOC may undergo a qualitative change.

## Conclusion

We demonstrate a new kind of He-radiation tolerant material, SiOC, based on TEM and proton backscattering spectrometry, as well as first-principles theory. Unlike crystalline solids, where implanted He becomes immobilized in nanometer-scale precipitates, He in SiOC remains in solution and outgasses from the material *via* atomic-scale diffusion without damaging its free surfaces. The rapid He outgassing in SiOC may result from small He interstitial formation and migration energies as well as negligible He dimer interaction energies. Our finding reveals a novel strategy to design He-damage resistant materials, which open a path to advancing the development of next generation nuclear-radiation resistant materials.

## Methods

### Sample synthesis

We grow SiOC samples by radio frequency (RF) co-sputtering of SiO_2_ (purity 99.5%) and SiC (purity 99.995%) onto thermally oxidzed Si (100) substrates for He^+^ implantation studies and carbon substrates for RBS compositional analysis at room temperature. Both targets were obtained from AJA International, Inc. The base pressure prior to deposition was 9.8 × 10^−6^ Pa and the partial pressure during Ar sputtering was ~0.65 Pa. The surface of the Si substrate contained a ~300 nm-thick layer a-SiO_2_ grown by thermal oxidation. The thickness of the deposited SiOC films was ~300 nm. The average composition determined by Rutherford backscattering spectrometry is Si_3_O_4_C_3_ (Supplementary Fig. S4).

### Ion implantation

Implantation was carried out using 50 keV He^+^ ions at room temperature (300 K) and at 600 °C (873 K) in a 140 kV accelerator under a base pressure better than 1.3 × 10^−4^ Pa. Target temperatures were maintained using a sample stage heater connected to a thermocouple attached to the target holder. The sample surface was inclined with respect to the ion beam at an angle of 45 degrees so that the majority of the He was implanted into the ~300 nm top surface layer. Depth profiles of implanted He were computed using SRIM^48^. Under our implantation conditions, a dose of 1.58 × 10^21^ ions/m^2^ gives rise to a peak He concentration of 10 atom%.

### Proton backscattering

The amount of retained He in our samples was measured using proton-based non-Rutherford backscattering (p-BS) with 2.44 MeV H^+^ ions^49^ in a modified General Ionex 1.7MV Tandetron accelerator. Backscattered H energy spectra were collected using a solid-state detector with 20 keV energy resolution mounted at a 160-degree backscattering angle. The depth sensitivity of this technique is greater than 1 μm, i.e. well in excess of the maximum depth of implanted He in our samples. However, since the depth resolution of p-BS is low, our measurements should be understood to show either the presence or absence of He, but not its distribution as a function of depth beneath the sample surface. The detection limit of the p-BS technique corresponds to a dose that yields a peak He concentration of 1 atom% in our implantation experiments.

### TEM characterization

The microstructure of our samples before and after ion implantation was characterized using a FEI Tecnai G2 F20 TEM. The operating voltage for this instrument was 200 kV. TEM specimens were prepared using a combination of mechanical grinding, dimpling, and polishing followed by low-energy (3.5 keV) Ar ion milling.

### *In-situ* implantation

The *in-situ* experiments were performed at the MIAMI-1 facility at the University of Huddersfield^31^. This consists a low energy, Colutron ion-accelerator coupled to a JEOL JEM-2000FX TEM in which the ions are incident on the specimen at 30° to the direction of the electron beam. The microscope was operated at 80 kV (in order to minimize electron-induced radiation damage) with the specimen horizontal in the TEM (i.e. its surface normal to the electron beam). Implantations were conducted using 3 keV He^+^ ions with a flux of 5 × 10^17^ ions/m^2^/s to a fluence of 2 × 10^21^ ions/m^2^ for implantation at room temperature and with a flux of 4.8 × 10^17^ ions/m^2^/s to a fluence of 3 × 10^21^ ions/m^2^ for an implantation using a liquid-nitrogen-cooled holder at its lowest temperature of 107 K.

### DFT calculations

Atomistic modeling was performed using the plane wave-based first-principles DFT code VASP^50^. We employed the Perdew-Burke-Ernzerhof (PBE)^51^ exchange-correlation functional within projector-augmented-wave approach^52^, a gamma-point only K point mesh, a 500 eV plane wave kinetic energy cutoff, and an energy convergence threshold of 10^−4^ eV for the electronic self-consistent loop. Standard pseudopotentials were used (Si, O, C, H, and He in VASP’s nomenclature). Ionic relaxations were performed using conjugate gradient energy minimization and a force convergence criterion of 10^−2^ eV/Å. Migration energy barriers were calculated *via* the climbing image nudged elastic band (NEB) method^53^ with a transition image separation distance of ~0.5 Å.

An orthorhombic, 192-atom a-SiO_2_ supercell with dimensions 14.33 × 14.33 × 14.33 Å was generated *via* the melting-and-quenching method using a ReaxFF classical potential^54^ and the LAMMPS code^55^—as described in a previous study^33^—and subsequently relaxed within VASP. Hydrogenated or H-free C-containing environments within SiOC were modeled by replacing two nearest-neighbor O atoms with two C-H complexes or two C atoms, respectively. He dopants were introduced as interstitial point defects within the simulation cell. Their formation energies, ${E}_{{Form}}$, were calculated as

1 $${E}_{{Form}}=E({material}+{He})-E({material})-E({He})$$

where $E({material})$ denotes the energy of the model prior to He insertion, $E({material}+{He})$ is its energy with one He interstitial at the site of interest, and $E({He})$ denotes the energy of a single He atom in vacuum. The interaction energy between two He interstitials, ${E}_{{Int}}$, is defined as

2 $${E}_{Int}=E(material+2He)+E(material)-{E}_{1}(material+He)-{E}_{2}(material+He)$$

where $E({material}+2{He})$ is the energy of the model with two He interstitials while ${E}_{1}({material}+{He})$ and ${E}_{2}({material}+{He})$ are the model energies containing the individual He atoms at their respective sites.

### Data availability

All data is available from the authors upon reasonable request.

## Electronic supplementary material

Supplementary information
