# Supplementary material for: Rapid and damage-free outgassing of implanted helium from amorphous silicon oxycarbide
Source: Sci Rep. 2018 Mar 22;8:5009. doi: 10.1038/s41598-018-23426-y (PMC5864747; doi:10.1038/s41598-018-23426-y)
Supplement: Supplementary file 1 — Supplementary information [file 41598_2018_23426_MOESM1_ESM.pdf]

Supplement file

Rapid and damage-free outgassing of implanted helium from amorphous silicon oxycarbide

Qing Su[1#], Hepeng Ding[2, 3#], Lloyd Price[4], Lin Shao[4], Jonathan A. Hinks[5], Graeme Greaves[5], Stephen E. Donnelly[5], Michael J. Demkowicz[2], and Michael Nastasi[1,6*]

[1]Nebraska Center for Energy Sciences Research, University of Nebraska-Lincoln, Lincoln, NE 68583-0857, USA

[2]Department of Materials Science and Engineering, Texas A&M University, College Station, TX 77843-3128, USA

[3]Department of Materials Science and Engineering, Massachusetts Institute of Technology, Cambridge, MA 02139, USA

[4]Department of Nuclear Engineering, Texas A&M University, College Station, TX 77843-3128, USA

[5]School of Computing and Engineering, University of Huddersfield, HD1 3DH, UK

[6]Department of Mechanical and Materials Engineering, University of Nebraska-Lincoln, Lincoln, NE 68583-0857, USA

[#] Co-first authors

*Corresponding author: (+1) 402-472-3852.

Email address: mnastasi2@unl.edu (Michael Nastasi)

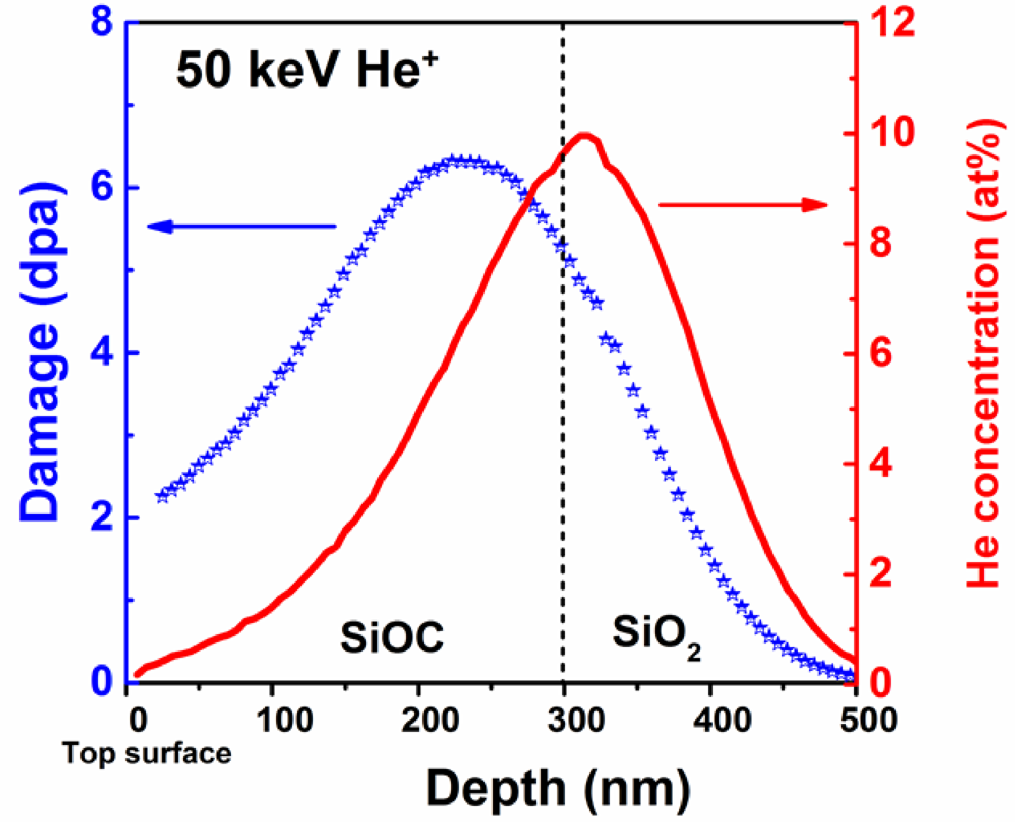

Fig. S1: The helium concentration and irradiation damage as function of penetrating depth simulated by TRIM (10 atom% peak, dose of 1.58×10^{21} ions/m^2). Besides implantation, there is, according to the simulation, approximately 6 dpa peak irradiation damage in SiOC films.

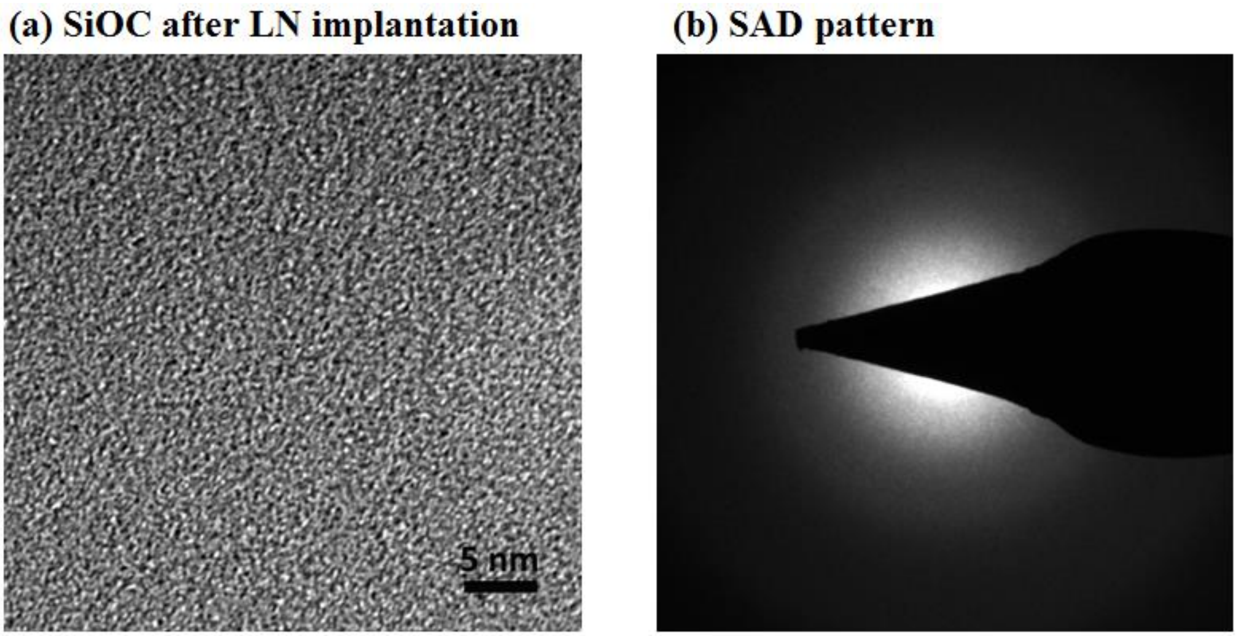

Fig. S2: (a) The high resolution TEM image of SiOC layer after 90 atom% helium implantation. No helium bubbles, phase segregation, void formation were observed. (b) The corresponding SAD pattern of SiOC layer after 90 atom% helium implantation, further confirming the materials remain its glassy state.

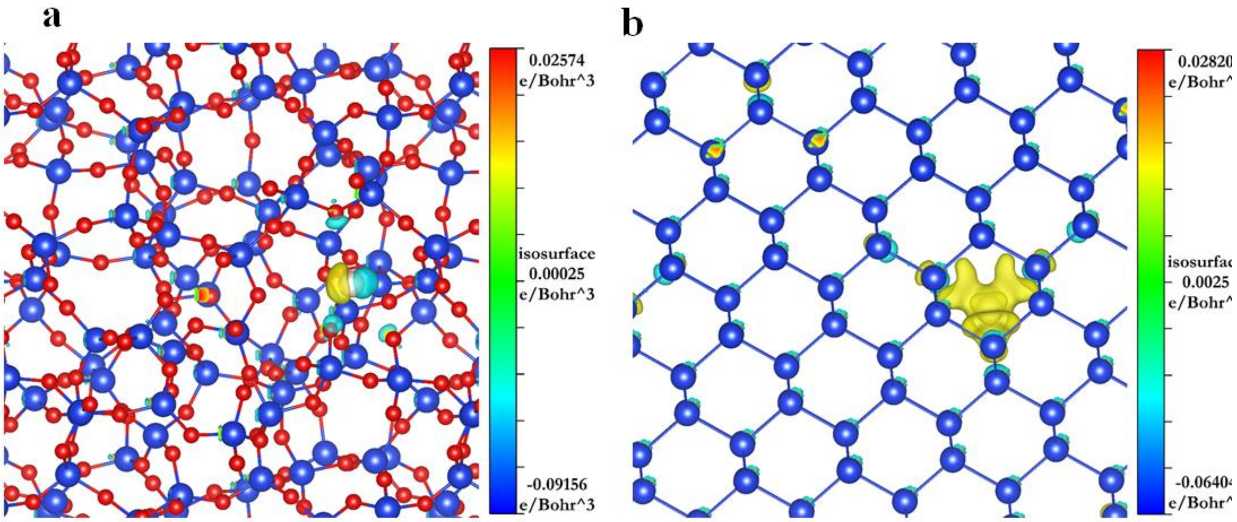

Fig. S3: Charge density difference plots caused by He interstitial in Si and SiO_2. (a) Si and (b) SiO_2. Much more charge density changes are visually observed in Si than in SiO_2, even though that the isosurface value used in Si is 10 times of that used in SiO_2.

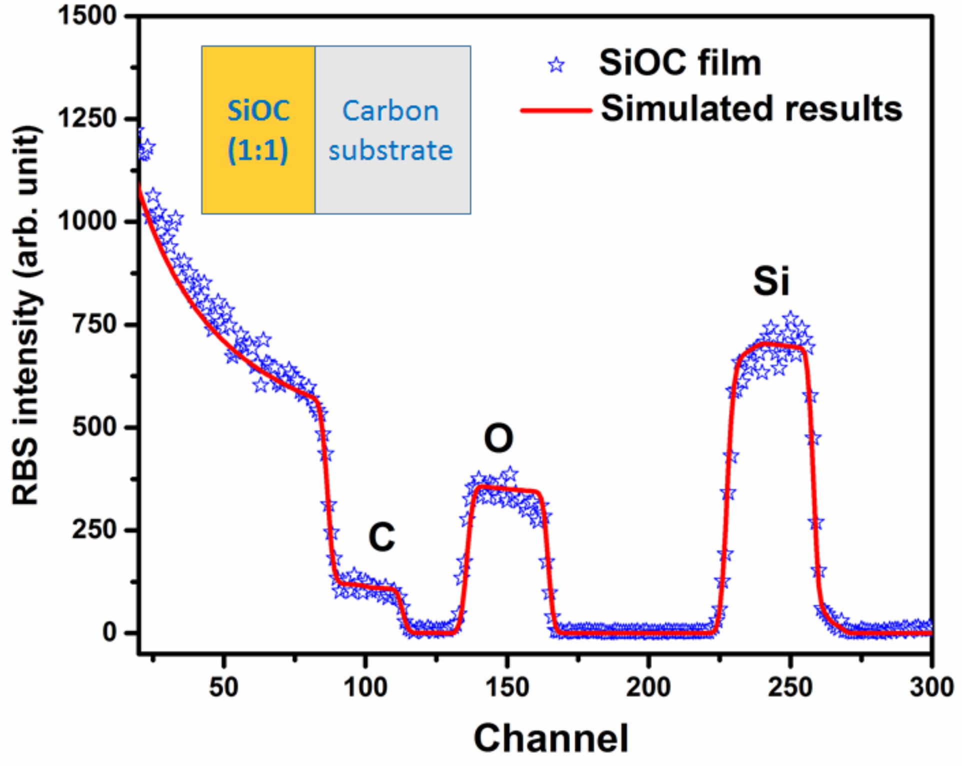

Fig. S4: RBS spectra of SiOC film on C substrate. The symbols represent the experimental data and the lines are RUMP fitting results which reveal that the nominal atomic composition of SiOC is Si-30%, O-40%, C-30%.
